# Regional gray matter density is associated with achievement motivation: evidence from voxel-based morphometry

**DOI:** 10.1007/s00429-012-0485-3

**Published:** 2012-12-05

**Authors:** Hikaru Takeuchi, Yasuyuki Taki, Rui Nouchi, Atsushi Sekiguchi, Yuka Kotozaki, Carlos Makoto Miyauchi, Ryoichi Yokoyama, Kunio Iizuka, Hiroshi Hashizume, Seishu Nakagawa, Keiko Kunitoki, Yuko Sassa, Ryuta Kawashima

**Affiliations:** 1 Smart Ageing International Research Center, Institute of Development, Aging and Cancer, Tohoku University, 4-1 Seiryo-cho, Aoba-ku, Sendai, 980-8575 Japan; 2 Division of Developmental Cognitive Neuroscience, Institute of Development, Aging and Cancer, Tohoku University, Sendai, Japan; 3 Japan Society for the Promotion of Science, Tokyo, Japan; 4 Department of Functional Brain Imaging, Institute of Development, Aging and Cancer, Tohoku University, Sendai, Japan; 5 Faculty of Medicine, Tohoku University, Sendai, Japan

**Keywords:** Achievement motivation, Voxel-based morphometry (VBM), Putamen, Orbitofrontal cortex (OFC), Regional gray matter density (rGMD), Precuneus

## Abstract

Achievement motivation can be defined as a recurrent need to improve one’s past performance. Despite previous functional imaging studies on motivation-related functional activation, the relationship between regional gray matter (rGM) morphology and achievement motivation has never been investigated. We used voxel-based morphometry and a questionnaire (achievement motivation scale) to measure individual achievement motivation and investigated the association between rGM density (rGMD) and achievement motivation [self-fulfillment achievement motivation (SFAM) and competitive achievement motivation (CAM) across the brain in healthy young adults (age 21.0 ± 1.8 years, men (*n* = 94), women (*n* = 91)]. SFAM and rGMD significantly and negatively correlated in the orbitofrontal cortex (OFC). CAM and rGMD significantly and positively correlated in the right putamen, insula, and precuneus. These results suggest that the brain areas that play central roles in externally modulated motivation (OFC and putamen) also contribute to SFAM and CAM, respectively, but in different ways. Furthermore, the brain areas in which rGMD correlated with CAM are related to cognitive processes associated with distressing emotions and social cognition, and these cognitive processes may characterize CAM.

## Introduction

Achievement motivation has many definitions; it can be defined as a recurrent need to improve one’s past performance (McClelland 1987) or as the desire to handle difficult tasks and succeed in them (Kurasawa 2001). Achievement motivation predicts better performance in various domains and has thus been well studied (Pang et al. 2009). It also predicts economic success and managerial ability at the workplace (McClelland 1961; McClelland and Boyatzis 1982). Furthermore, it predicts superior academic performance, better quality of learning, and increased persistence and effort in studies (Deci et al. 1996; O’Connor et al. 1966; Ryan and Deci 2000).

Some psychological studies have investigated associations between the type of achievement motivation and a wide range of situations and conditions, including factors such as task difficulty and individual ability (Nicholls 1984). Most have indicated that self-evaluative processes and one’s perceived social self are important in this respect (Nicholls 1984). In terms of measurement of the general achievement motivation of an individual (as a trait or as a trait-related concept), a number of questionnaires have separated achievement motivation and its related concepts into two categories: motivation independent of others and motivation dependent on the views of others or society. For example, the California Psychological Inventory separates achievement tendency into Achievement via Conformance and Achievement via Independence (Gough and Bradley 1996), and the Need achievement scale (Bendig 1964) measures personal need achievement and social need achievement. Horino and Mori developed an achievement motivation scale in the form of a questionnaire that evaluates two psychometrically derived achievement motivations: self-fulfillment achievement motivation (SFAM) and competitive achievement motivation (CAM) (Horino and Mori 1991). This scale evaluates two psychometrically derived achievement motivations: self-fulfillment achievement motivation (SFAM) and competitive achievement motivation (CAM). SFAM is achievement motivation directed at pursuing goals evaluated by one’s own standards of achievement regardless of the values of others and society. CAM is achievement motivation directed at seeking social prestige by defeating others and achieving better results than they achieve. People with higher SFAM scores use a wider range of learning strategies (Yamada et al. 2010). Higher SFAM also leads to higher self-efficacy, whereas higher CAM tends to lead to lower self-esteem (Shao and Daibo 2008) and is associated with a state of enervation (Horino and Mori 1991). SFAM is negatively correlated with feelings of inferiority (Yamada and Mizuno 2011). CAM is positively correlated with a competitive spirit, and both SFAM and CAM are negatively correlated with a lack of interest in learning (Ota 2005b). SFAM is negatively correlated with apathy toward classes and life at university (Fukuoka 2000). SFAM is positively correlated with a tendency to think highly of one’s efforts in competitive situations (Ota 2005a). These data suggest the validity of this questionnaire. Thus, although, this questionnaire has been only used in Japanese study populations, not only the scientific validity of the questionnaire is not undermined by that at all, also the basic concept of the separation of achievement motivation directed by one’s own values and the values of society/others is common to other generally used measures.

Previous neuroimaging studies revealed the neural basis of motivation induced externally by monetary reward. These studies consistently revealed the involvement of the orbitofrontal cortex (OFC) and striatum, as well as other regions, in motivation (Breiter et al. 2001; Elliott et al. 2004; Kirsch et al. 2003; Knutson et al. 2000). Furthermore, another functional imaging study (Mizuno et al. 2008) revealed neural activity related to academic achievement motivation, which is achievement motivation in academic areas. The researchers observed brain activity under experimental conditions designed to induce academic achievement motivation. These manipulations involved a sense of competence and achievement, which are known to cause higher academic achievement motivation (Maehr 1984). Part of the striatum, the putamen, was found to be more active when subjects were highly motivated to learn than under control conditions, and individual activity in this area was positively correlated with the trait of academic achievement motivation measured using a questionnaire (Mizuno et al. 2008).

Despite studies of functional activation in association with externally modulated motivation and of the effects of achievement motivation on functional activity, the gray matter (GM) structural bases underlying individual differences in achievement motivation have never been investigated. Furthermore, the neural bases of achievement motivation that is not limited to academic areas and accounting for achievement motivation according to one’s own values as well as the values of society/others have never been investigated.

Based on previous psychological and brain imaging studies, we developed two a priori hypotheses (A) and (B). (A) We hypothesized that regional GM (rGM) structures of OFC and the striatum, together with other regions that have been associated with motivation or academic achievement motivation per se, are associated with the two achievement motivation categories of SFAM and CAM. (B) Furthermore, we assumed CAM as achievement motivation on the basis of associations with others and society. Thus, we hypothesized that brain structures related to the social cognition network is associated with CAM. This network was revealed by previous neuroimaging studies that investigated brain activity when considering the perspectives of others. The social cognition network is also involved in a series of social cognitions such as theory of mind and involves regions such as the medial prefrontal cortex (mPFC), superior temporal sulcus, and posterior cingulate cortex (PCC) [for a summary of the neural circuitry involved in social cognition, see Pelphrey and Carter (2008)]. These structures would also be associated with CAM.

For the purpose of testing these two hypotheses and reveal the GM structural bases underlying individual achievement motivation, we investigated how individual differences in achievement motivation were associated with rGM density (rGMD) using voxel-based morphometry (VBM) (Good et al. 2001b). The two types of achievement motivation were assessed using an achievement motivation scale in the form of a questionnaire (Horino and Mori 1991).

Achievement motivation is a driving force for achievement across a wide range of areas in everyday life. In particular, it is well known that achievement motivation is important in learning and education (Atkinson and Feather 1964). Psychological studies have tended to focus on factors predictive of individual success in academic areas and societies, including intelligence, creativity, and emotional intelligence (Colom et al. 2006; Haier et al. 2004; Jung et al. 2010; Takeuchi et al. 2011a, c, g, 2012a), but further study is required to determine the neural mechanisms underlying achievement motivation.

Both functional imaging and structural studies have advantages and disadvantages, and the two approaches complement each other (Takeuchi et al. 2011a, g). Structural imaging studies are particularly useful for investigating the anatomical correlates of personal characteristics involving a wide range of behaviors and ideas that occur outside the laboratory, such as achievement motivation. This is because unlike the results of functional magnetic resonance imaging (fMRI) studies, the results of structural imaging studies (a) are not limited to the specific regions engaged in the task or stimuli during scanning and (b) do not have to take into account unusual situations mimicking activities in daily life (Okamoto et al. 2004). Furthermore, while fMRI studies have certain apparent advantages, one disadvantage is that simplified everyday tasks have to be performed in an MRI scanner because of the constraints of MRI, and this may lead to different activation patterns in the brain (Okamoto et al. 2004).

## Methods

### Subjects

One hundred and eighty-five healthy, right-handed individuals (94 men and 91 women; age, 21.0 ± 1.8 years) participated in this study as part of our ongoing project investigating the associations among brain imaging, cognitive functions, aging, genetics, and daily habits (Takeuchi et al. 2010b, 2011a, b, c, f; Taki et al. 2010, 2011). Data from subjects in this study are to be used in other studies irrelevant to the theme of this study. Some of the subjects who participated in this study also became subjects of intervention studies (psychological and imaging data recorded before the interventions were used in this study). Psychological tests and MRI scans not described in this study were performed together with those described in this study. All subjects were university, college, or postgraduate students or subjects who had graduated from such institutions within 1 year before the experiment; all had normal vision. No subject had a history of neurological or psychiatric illness. Handedness was evaluated using the Edinburgh Handedness Inventory (Oldfield 1971). Written informed consent was obtained from each subject in accordance with the Declaration of Helsinki (1991). This study was approved by the Ethics Committee of Tohoku University.

### Achievement motivation scale

The Japanese version of the achievement motivation scale (Horino and Mori 1991) was used to assess individual achievement motivation. This questionnaire is a self-report measure of achievement motivation. A more detailed discussion of how it was developed can be found in a previous study (Horino and Mori 1991). This questionnaire includes 23 items and employs a 7-point Likert scale with a response format ranging from “not true of me at all” to “very often true of me”. Responses of the subjects render composite scale scores for two factors: SFAM and CAM.

It has been increasingly stressed that achievement motivation comprises multiple factors, and Horino and Mori (1991) identified the above two factors of achievement motivation via factor analysis.

The following are examples of items in this questionnaire:

(1) “I always want to have some goals. (SFAM)”; (2) “It is more important to do my best in my own way than to beat others (SFAM)”; (3) “I cannot stand being defeated by opponents. (CAM)”; and (4) “Success is getting fame and high-ranking positions (CAM)”.

These factors were identified on the basis of data from 236 subjects (Horino and Mori 1991). The number of items used for assessing SFAM and CAM may seem too small to produce reliable data. However, the reliability of the questionnaire can be estimated by, for example, Spearman–Brown reliability coefficients, which are determined by both the length of the questionnaire and the strength of the correlations among the items or factors it includes. For the current questionnaire, the Spearman–Brown reliability coefficients for SFAM and CAM were 0.87 and 0.91, respectively (Horino and Mori 1991). Considering that a Spearman–Brown reliability coefficient of >0.70 is considered reliable (Nunnally 1978), we believe our data for these two factors are very reliable.

### Assessment of psychometric measures of general intelligence

Raven’s Advanced Progressive Matrix (RAPM; Raven 1998) was used to assess intelligence (Raven 1998) and adjust for the effect of general intelligence on brain structures (Haier et al. 2004). For more details on how RAPM was administered, refer to our previous study (Takeuchi et al. 2010a, b).

### Assessment of personality

Personality was assessed using the NEO Five-Factor Inventory (NEO-FFI) (Costa and McCrae 1992). NEO-FFI is a self-administered measure of normal personality functioning and produces summary scores across five domains: neuroticism, extraversion, openness, agreeableness, and conscientiousness. This measure was used to investigate the criterion-related validity as well as discriminant validity of the achievement motivation scale.

### Image acquisition and analysis

All MRI data acquisition was performed using a 3-T Philips Intera Achieva scanner. High-resolution T1-weighted structural images (T1WIs; 240 × 240 matrix, TR = 6.5 ms, TE = 3 ms, FOV = 24 cm, slices = 162, slice thickness = 1.0 mm) were collected using a MPRAGE sequence.

### Preprocessing of T1-weighted structural data

Preprocessing of T1WI data was performed using VBM2 software (Gaser 2007), an extension of SPM2. Default parameter settings were used (Gaser 2007). As summarized in our previous study (Takeuchi et al. 2011a), potential correlates of rGM structures may include the number and size of neurons and glial cells, the level of synaptic bulk, and the number of neurites (May and Gaser 2006), although this notion remains to be proven by histological studies. rGMD is known to be associated with various cognitive abilities, and investigation of these associations can identify the brain regions associated with specific cognitive abilities or characteristics (Frangou et al. 2004; Takeuchi et al. 2011a; Tisserand et al. 2004).

We used a scanner-specific customized GM anatomical template and prior probability maps of GM and white matter (WM) images created from T1WIs taken using the same scanner employed in our previous study (Takeuchi et al. 2010b). This action was taken because the contrast of T1WIs obtained in this study might have differed from the existing template and because each scanner introduces specific non-uniformities in image intensity and inhomogeneities in the B0 field. The subjects in this previous study and those in the present study have equivalent characteristics, and all participated in the above-mentioned project. Other than that, thereafter, each subject’s image was segmented and normalized using VBM2’s normal procedure (segmentation: cross-sectional data, 1 time point per subject). Subsequently, all images were smoothed by convolving them with an isotropic Gaussian Kernel of 10-mm full width at half maximum (FWHM). The resulting maps representing rGMD were then forwarded for multiple regression analyses. We did not modulate resulting images and hence the images represent rGMD. When we use rGMD, VBM can be thought of as a comparison between the relative concentrations of gray or white matter structures in the spatially normalized images. When we use regional gray matter volume, VBM can be thought of as comparing the absolute volume of gray or white matter structures. The results of rGMD and regional gray matter volume are usually similar (Good et al. 2001a). To our knowledge, the implication of the (subtle and probably statistically meaningless) difference between these two analyses is not known. Each of these methods has been frequently used in structural studies (Mechelli et al. 2005). In the present study, we used rGMD because our study which measured structural correlates of relevant cognitions used rGMD (the reasons, why this previous study used rGMD can be seen in Takeuchi et al. 2011a).

VBM2 was used instead of VBM5 or VBM8 for the preprocessing of T1-weighted structural imaging data because T1WIs obtained using the above-mentioned MPRAGE sequence were incompatible with preprocessing with VBM5/SPM5 and VBM8/SPM8. When VBM5 or SPM5 was used, many apparent segmentation errors occurred, unlike when the optimized protocol of VBM2 was used. Segmentation errors apparent at first glance were not found when VBM2 or VBM8 was used. However, when VBM8 was used, the test–retest reliability of total GMV of 50 subjects who participated in a 1-week longitudinal intervention study in which T1WI was taken on the first day of the experiment and 1 week thereafter (Takeuchi et al. 2011d) was 0.746, whereas when VBM2 was used, the reliability was 0.980. These procedures (preprocessing with VBM2 and statistical analyses using different versions of SPM/VBM) were also used in previous studies (Ilg et al. 2008; Takeuchi et al. 2010b, 2011e, 2012d). Although the above-mentioned data do not indicate that preprocessing with VBM5/VBM8 is worse, it does say something about the compatibility between T1WIs of certain sequences and VBM5/VBM8. One possible reason for the discrepancy between the outcomes obtained with VBM2 and VBM5/VBM8 is the use of tissue probability maps. VBM5 and VBM8, unlike VBM2, do not utilize tissue probability maps. As per our understanding, the segmentation error is not due to any problem with the data obtained (if that was the case, the segmentation would also be problematic when VBM2 was used). When newer versions of VBM were used, T1WIs taken using another 1.5 T scanner (Takeuchi et al. 2012c) and the magnetization transfer ratio images (Takeuchi et al. 2011c) taken using the same scanner used in the present study did not result in disastrous segmentation. Compared with these images, the T1WIs used in this study appeared clearer, but the contrasts among GM, WM, and CSF were substantially different. This may or may not have been the cause of the error.

The automated preprocessing procedure is the fundamental limitation of VBM. As described in our previous study (Takeuchi et al. 2011f), while automated preprocessing with VBM procedures allowed the processing of large amounts of data at the whole-brain level without limiting the choice of regions to a priori hypothesized selections, the procedures may suffer from more preprocessing errors compared with manual volumetry (Kennedy et al. 2009). We accepted this trade-off.

### Statistical analyses

We investigated rGMD associated with individual differences in SFAM and CAM. Statistical analyses of imaging data were performed with SPM8.

In rGMD analysis, we included only voxels that showed GM values of >0.05 in all subjects. The primary purpose for using a GM threshold is to cut the periphery of the GM area and effectively limit the area for analysis. We performed this procedure by limiting the area for analysis to areas likely to be GM. Voxels outside the brain areas are more likely to be affected by signals outside the brain through smoothing. Masking the analysis to brain areas is performed by default during fMRI analyses with SPM. The GM value threshold of 0.05 is a widely used value that has been reported in numerous previous VBM studies (Beal et al. 2007; Focke et al. 2008; Mueller et al. 2006; Nauchi and Sakai 2009; Schaufelberger et al. 2007; Takeuchi et al. 2010b, 2011a; White et al. 2003).

In whole-brain multiple regression analyses, we tested for a relationship between SFAM and CAM as measured by the achievement motivation scale scores and rGMD. The analyses were performed considering sex, age, RAPM score, and total brain volume (total GM volume + total WM volume) as additional covariates, resulting in six covariates as a whole in this multiple regression analysis. Total brain volume was used instead of intracranial volume because the above-mentioned preprocessing methods did not classify CSF and other non-brain tissues well. For each covariate, the “overall mean” option was used for centering.

We did not include personality variables as covariates in the whole-brain imaging analyses because we assume that these basic personality variables cover a wide range of cognitive modules, some of which would be fundamentally shared by the achievement motivation measures used in this study. Thus, these effects should not be regressed out. For example, the agreeableness factors of NEO-FFI and CAM both have apparent pro- and anti-social components (in case of agreeableness, pro-sociality and in case of CAM, the opposite). When investigating the anatomical correlates of CAM, this pro-social component should not be regressed out because it is in fact exactly what we would like to reveal.

Next, we investigated whether the relationships between rGMD and the SFAM score or those between rGMD and the CAM score differed between sexes (whether the interaction between sex and the SFAM score or that between sex and the CAM score affected rGMD). In whole-brain analyses, we used a voxel-wise analysis of covariance (ANCOVA) in which sex difference was a group factor (using the full factorial option of SPM8). In this analysis, age, RAPM score, total brain volume, SFAM score, and CAM score were covariates. All of these covariates, except for total brain volume, were modeled so that each covariate’s unique relationship with rGMD could be seen for each sex (using the interactions option in SPM8), which would allow the interaction effects of sex and the covariates to be investigated. The interaction effect between sex and the SFAM score on rGMD as well as that between sex and the CAM score on rGMD were assessed using t-contrasts.

A multiple comparison correction was performed using the voxel-level family-wise error (FWE) approach at the whole-brain level. The statistical significance level was set at *P* < 0.05, corrected for multiple comparisons.

In rGMD analysis, for areas with a strong a priori hypothesis, namely the bilateral striatum and OFC, the statistical significance level was set at *P* < 0.05, with a small volume correction for multiple comparisons (voxel-level FWE) in regions of interest (ROIs). As described in “Introduction”, these ROIs were chosen because they played central roles in previous studies of functional activation associated with externally modulated motivation and the activity of the striatum correlated with academic achievement motivation. All ROIs were constructed using the WFU PickAtlas Tool (http://www.fmri.wfubmc.edu/cms/software#PickAtlas) (Maldjian et al. 2003, 2004). ROI of OFC was obtained by adding the mask images of the bilateral orbital gyri in the Talairach Daemon (Lancaster et al. 2000) option of the PickAtlas. ROI of the striatum was obtained by adding the mask images of the bilateral caudates and bilateral putamens in the Automated Anatomical Labeling option (Tzourio-Mazoyer et al. 2002) of the PickAtlas.

## Results

### Behavioral data

Table 1 shows the average age plus standard deviation (SD) as well as the scores plus SD for RAPM, SFAM, and CAM in males and females. Table 2 shows the distributions of SFAM and CAM scores in males and females. Females showed significantly higher SFAM scores than males (*P* = 0.016, *t* = 2.42, two-tailed *t* test). Males showed significantly higher CAM scores (*P* = 0.001, *t* = 3.26, two-tailed *t* test). Simple correlation analysis revealed that SFAM and CAM scores were significantly and positively correlated (*r* = 0.24; *P* < 0.001; *t* = 3.37).

**Table 1:** Demographic variables of the study participants

Measure	Males		Females	
	Mean	SD	Mean	SD
Age	20.81	1.86	21.16	1.74
RAPM	28.81	3.26	28.30	3.68
SFAM	67.21	9.35	70.41	8.56
CAM	53.04	7.83	49.04	8.84

**Table 2:** Distribution of SFAM and CAM scores of the study participants

	20–29	30–39	40–49	50–59	60–69	70–79	80–89	90 and above
SFAM (male)	0	0	3	16	33	34	7	1
SFAM (female)	0	0	1	9	30	38	12	1
CAM (male)	0	5	24	46	17	2	0	0
CAM (female)	2	8	40	29	12	0	0	0

Table 3 shows the simple correlation coefficients for the SFAM/CAM scores and personality variables in NEO-FFI. The SFAM and CAM scores were weakly (*r* < 0.4) correlated with various personality subscales in NEO-FFI. The weak correlation clearly demonstrated the discriminant validity of the scale. Furthermore, both SFAM and CAM scores were significantly positively correlated with extraversion, which is partly defined by how active and energetic an individual is (Costa and McCrae 1992). On the other hand, only CAM scores were found to have a significant negative correlation with agreeableness, which is partly characterized by a type of pro-sociality, such as altruism (Costa and McCrae 1992). Finally, SFAM scores were significantly positively correlated with conscientiousness, which includes achievement as well as several other factors (Costa and McCrae 1992). These findings demonstrated the criterion-related validity of the scale.

**Table 3:** Simple correlation coefficients (*P* values) of the simple correlation between SFAM/CAM scores and personality variables in NEO-FFI

	Neuroticism	Extraversion	Openness	Agreeableness	Conscientiousness
SFAM	−0.039 (0.596)	0.374 (1.61 × 10^−7^)	0.247 (0.001)	0.255 (4.45 × 10^−4^)	0.309 (1.93 × 10^−5^)
CAM	0.069 (0.353)	0.147 (0.046)	−0.175 (0.017)	−0.171 (0.020)	0.110 (0.138)

### Correlation between rGMD and the SFAM score on the achievement motivation scale

We investigated rGMD associated with individual differences in SFAM. Multiple regression analysis considering age, sex, general intelligence, total brain volume, and CAM revealed that the SFAM score on the achievement motivation scale was not significantly correlated with rGMD in any of the regions (*P* > 0.05 corrected for multiple comparisons at voxel-level FWE at the whole-brain level).

**Fig. 1:**
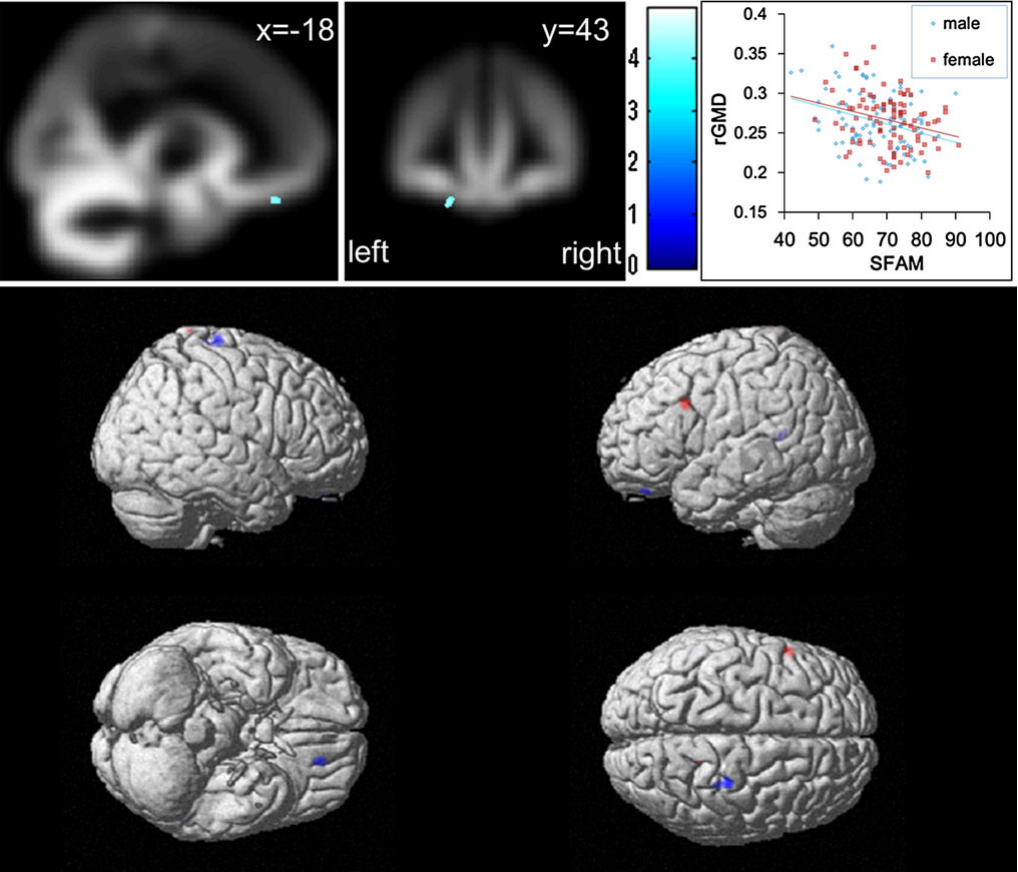
Anatomical correlates of SFAM. In the *left panel*, the region of correlation is overlaid on the mean smoothed rGMD image from all participants. rGMD was correlated with individual SFAM in the left OFC. Results are shown with *P* < 0.0001 uncorrected for visualization purposes. Note that the cluster may look small compared with the extent of the significant correlation described in the “Results” section because the thresholds used in SVC and in this figure were different; a different threshold was used here to ensure that a common threshold was applied for all figures. As shown in the scatterplot, the voxels show the rGMD values that are considerably higher than the threshold for grey tissue probability. The *intensity of the color* represents the *T* score. The *right panel* shows a scatter plot of the relationship between the SFAM score and mean rGMD values of the significant cluster in OFC. The *blue line* represents the regression line for males, and the *red line* represents the line for females. The *panel below* shows the tendencies of the positive (*red*)/negative (*blue*) correlations between rGMD and SFAM. The results are shown with *P* < 0.001 uncorrected and extent threshold of 100 voxels. The particulary strong tendencies were seen around the postcentral gyrus (negative) and the left lateral prefrontal cortex (positive). The region of correlation between SFAM and rGMD seems to lie slightly outside the brain in the *panel below*. However, this may be due to the use of a custom template. As can be seen in the *top left panel*, the region of correlation lies well within the GM area. Furthermore, the scatterplot shows that the average cluster rGMD value is well >0.25, confirming this notion

Among areas with a strong a priori hypothesis, SVC was employed and significant negative correlations were found in OFC (*x*, *y*, *z* = −20, 43, −27, *t* = 4.21, SVC for areas with a strong a priori hypothesis: *P* = 0.003 corrected for multiple comparisons at voxel-level FWE, 163 voxels with *P* < 0.05, corrected; Fig. 1). No other significant results were observed. However, there was some tendency toward a negative correlation in the region around the precentral and postcentral gyri (*x*, *y*, *z* = 31, −25, 72, *t* = 4.87, *P* = 0.060 corrected for multiple comparisons at voxel-level FWE at the whole-brain level), which is the node of the somatic marker circuitry, and a positive correlation in the left lateral prefrontal cortex (*x*, *y*, *z* = −54, 15, 32, *t* = 4.61, *P* = 0.151 corrected for multiple comparisons at voxel-level FWE at the whole-brain level). Thus, there might be other regions associated with SFAM, but this was not statistically confirmed by the present findings. These tendencies are shown together with other tendencies (*P* < 0.001, uncorrected, 100 voxels) in Fig. 1.

### Correlation between rGMD and the CAM score on the achievement motivation scale

**Fig. 2:**
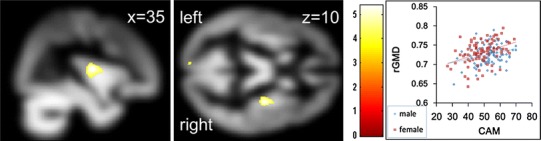
Anatomical correlates of CAM. In the *left panel*, the region of correlation is overlaid on the mean smoothed rGMD image from all participants. rGMD was correlated with individual CAM in an anatomical cluster that included the right insula and right putamen. Results are shown with *P* < 0.0001 uncorrected for visualization purposes. The *intensity of the color* represents the *T* score. The *right panel* shows a scatterplot of the relationship between the CAM score and mean rGMD values of the significant cluster in this area. The *blue line* represents the regression line for males, and the *red line* represents the line for females

**Fig. 3:**
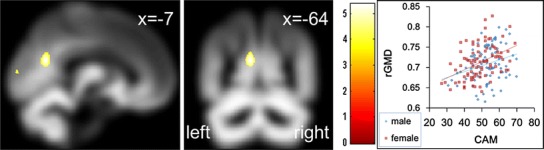
Anatomical correlates of CAM. In the *left panel*, the region of correlation is overlaid on the mean smoothed rGMD image from all participants. rGMD was correlated with individual CAM in the precuneus. Results are shown with *P* < 0.0001 uncorrected for visualization purposes. The *intensity of the color* represents the *T* score. The *right panel* shows a scatterplot of the relationship between the CAM score and mean rGMD values of the significant cluster in the precuneus. As shown in the scatterplot, the voxels show the rGMD values that are considerably higher than the threshold for grey tissue probability. The *blue line* represents the regression line for males, and the *red line* represents the line for females

We next investigated rGMD associated with individual differences in CAM. Multiple regression analysis considering age, sex, general intelligence, total brain volume, and SFAM revealed that the CAM score on the achievement motivation scale was significantly and positively correlated with rGMD in an area that spread across the right posterior insula and right putamen (*x*, *y*, *z* = 35, −11, 10, *t* = 5.37, *P* = 0.008 corrected for multiple comparisons at voxel-level FWE at the whole-brain level, 107 voxels with *P* < 0.05, corrected; Fig. 2) as well as an area in the precuneus (*x*, *y*, *z* = −7, −64, 23, *t* = 5.30, *P* = 0.010 corrected for multiple comparisons at voxel-level FWE at the whole-brain level, 100 voxels with *P* < 0.05, corrected; Fig. 3).

Among areas with a strong a priori hypothesis, SVC was employed. No other significant results were observed.

### Interaction effects between sex and the SFAM score on rGMD and those between sex and CAM on rGMD

The analysis of covariance using data from both sexes revealed the absence of interaction effects between the SFAM score and sex on rGMD. The analysis of covariance using data from both sexes revealed the absence of interaction effects between the CAM score and sex on rGMD. This result indicates absence of evidence to prove the existence of sex-specific relationships between achievement motivation and rGM structures.

## Discussion

To the best of our knowledge, this is the first study to reveal the anatomical bases underlying two aspects of achievement motivation: SFAM and CAM. Consistent with our hypotheses, achievement motivation was related to rGMD in OFC and the striatum and CAM was related to rGMD in the precuneus, which is the key node of the social cognition network. Our results showed that individual SFAM is related to rGMD in OFC. Furthermore, CAM is related to rGMD in the right putamen, right insula, and precuneus. These results suggest that the brain areas that play central roles in externally modulated motivation (OFC and the putamen, as described in “Introduction”) also contribute to SFAM and CAM, respectively, but in different ways. Furthermore, the brain areas in which rGMD correlated with CAM are related to cognitive processes associated with distressing emotions and social cognition, and these cognitive processes may characterize CAM.

We associated lower rGM in the precuneus and OFC with better functioning of these regions and larger rGM in the area around the putamen with better functioning of that region for the following reasons. First, in the areas around mPFC and OFC, increased functioning related to social and self-related components may be associated with decreased rGM structures (or increased cortical thinning) in young adults. This was also discussed in our previous studies (for details, see Takeuchi et al. 2011a, 2012b), but the same may hold true for the medial parietal area (precuneus). In these regions, developmental cortical thinning, which is probably caused by synaptic pruning (Sowell et al. 2003), is observed after adolescence, and advanced development of neural systems, which may well be related to matured cognitive abilities in most cases, is characterized by advanced cortical thinning and reduced rGM. A summary of the association between decreased rGM and increased functioning in these areas can be found in the review by Kanai and Rees (2011). However, in this context, this mechanism (less rGMV represents advanced development and better functioning of the region) can be applied to the area where a developmental decrease in rGM is observed until the age of the sample of this study (21 years). However, a number of widespread regions around the posterior superior temporal gyrus show age-related increases (instead of decreases) in rGM until approximately at 30 years of age (Sowell et al. 2003). Although the age-related change in the posterior insula that occurs at approximately 20 years of age was not confirmed in the study by Sowell et al., considering the proximity of the posterior insula and posterior temporal gyrus and the extent of the areas that showed the same age-related patterns of change around these regions in the lateral brain areas, we believe it is reasonable to assume that the posterior insula shows the same pattern of age-related change at approximately 20 years of age. Moreover, in our previous study, increased rGM in the superior temporal sulcus, which is one of the key nodes of the social cognition network, was associated with increased interpersonal emotional intelligence (Takeuchi et al. 2011a). Therefore, in our present study, we interpreted the increase in rGM in the posterior insula and putamen as reflecting a facilitation of the functioning of these regions. Although the association between reduced rGM and better cognitive function is not congruent with some clinical findings that show deteriorated cognitive functioning with reduced rGM, these mechanisms may well be caused by different physiological mechanisms such as neuronal degeneration, including neuron loss. These could lead to deficits of competencies and reduced GM signaling in VBM (Takeuchi et al. 2011a). In addition, note that these discussions related to cortical thinning and development are not necessarily restricted by the real age of subjects because of individual differences in these physiological phenomena among individuals of the same age (Hill and Frith 2003).

OFC is critically involved in the reward and motivation system in the brain, and highly developed function in this area may lead to higher SFAM. OFC is anatomically connected to a wide range of regions. It obtains sensory information from regions such as the gustatory and temporal cortices, affective information from the amygdala, and motivational information from the hypothalamus (Wallis 2007). Although many cognitive processes are attributed to functional activation in this region, the basic principle of this region’s role is considered to be the integration of such information to derive the value of potential reward outcomes and make that value available for activities such as effective decision making and reward-related learning (Wallis 2007). The region is considered critical in reward, hedonic experience, and motivation (Kringelbach 2005). Perhaps, decreased GM structure in this region, which is characterized by cortical thinning, may help one better integrate a wider range of information for deriving the value of potential reward outcomes and thus leading to higher autonomic motivation or SFAM. Consistent with this notion are the symptoms caused by OFC lesions. One of the most common symptoms of frontal lobotomy that involves damage to OFC is apathy, which may well be related to lower achievement motivation (Dougherty and Rauch 2007).

The putamen and insula are known to be involved in reward and cognitive processes associated with distressing emotions. More GM structures in these regions may lead to higher CAM through augmentation of motivation underlying these cognitive processes. The putamen is involved in many aspects of reward including reward magnitude (Cromwell and Schultz 2003), reward expectation (O’Doherty et al. 2002), and reward predictability (O’Doherty et al. 2003). Activation of the putamen is associated with motivation regardless of whether this is achievement or extrinsic motivation is caused by a monetary reward; the right putamen is more active when subjects are highly motivated to learn and when a monetary reward is given. Higher individual activity of the putamen is associated with high academic motivation (Mizuno et al. 2008). Furthermore, the insula and putamen are both involved in some form with emotions. Both these regions are active in response to hatred (Zeki and Romaya 2008) as well as romantic love (Bartels and Zeki 2004); they have also been implicated in disgust (Phillips et al. 1997) and anger (Paulus et al. 2010; Schultheiss et al. 2008). These emotions, including romantic love, are all distressing (Zeki and Romaya 2008). The putamen is also suggested to be involved in action or action planning related to these distressing emotions and reward (action planning driven by these distressing emotions and reward) (Haruno and Kawato 2006; Zeki and Romaya 2008). Finally, the recognition and experience of disgust is lost with damage to the posterior insula (Phillips et al. 1997). Together, these findings indicate that the insula and putamen are associated with distressing emotions and that the putamen is associated with processes related to a wide range of rewards and distressing emotions. CAM includes a contentious component that may well be related to anger, hatred, and disgust (defeating others and seeking social prestige by achieving better results than they achieve). To confirm this notion, we performed two multiple regression analyses using the psychological data of the subjects included in this project. In these analyses, age, sex, and RAPM, SFAM, and CAM scores were independent variables. The trait anger score on the State-Trait Anger Expression Inventory (STAXI) (Spielberger et al. 1999) was the dependent variable in one analysis and the 1-week state anger-hostility subscale of the short version of the profile of mood states (POMS) (McNair et al. 1992; Yokoyama 2005) in the other analysis. The former multiple regression analysis revealed that the CAM score was significantly and positively moderately correlated with the trait anger score on STAXI (*P* = 0.00001, *t* = 4.54), whereas the SFAM score was not (*P* = 0.155, *t* = −1.43). The latter multiple regression analysis revealed that the CAM score was significantly and positively weakly correlated with the 1-week state anger-hostility subscale of POMS (*P* = 0.01, *t* = 2.61), whereas the SFAM score was not (*P* = 0.254, *t* = −1.14). Perhaps, more GM structures are related to the augmentation of these functions, which in turn leads to increased drive that may underlie higher CAM. Partly congruent with this notion, a previous study (Schultheiss et al. 2008) showed that individuals with high implicit motivation (motivational dispositions that operate outside people’s conscious awareness and that orient, select, and energize spontaneous forms of behavior) show increased activation in a region around the posterior insula and putamen during recognition of facial expressions of anger and surprise.

Larger rGMD in the precuneus in subjects with higher competitive achievement may be a reflection of compromised pro-social cognitive abilities or characteristics, which in turn may make the nature of their achievement motivation competitive. Here, larger rGMD was associated with increased CAM in the precuneus. In addition, a tendency for a positive correlation between rGMD and CAM in mPFC was observed (*x*, *y*, *z* = 21, 51, 21, *t* = 4.04, 670 voxels with *P* < 0.001, uncorrected). The regions of the network to which mPFC and the precuneus belong are involved in diverse forms of social cognition that include theory of mind and the recognition of another’s perspective (Amodio and Frith 2006; Buckner et al. 2008). Numerous studies have shown that the precuneus is involved in perspective taking (for review, see Cavanna and Trimble 2006). mPFC and the precuneus are active when identifying the emotional states of others, and higher empathy is associated with greater activity in these regions (Schulte-Rüther et al. 2011). Furthermore, the regions around the precuneus and the ventral part of mPFC show greater activation when cooperation is required instead of competition (Decety et al. 2004). As described above, as in these medial prefrontal and medial parietal regions, increased cortical thinning (or less GM structure) seems to be associated with social and emotional cognitive abilities. Thus, increased rGMD in these regions in subjects with higher CAM may be a reflection of their compromised pro-social cognitive abilities or characteristics. These cognitive characteristics in turn may characterize their CAM, which seeks to surpass and defeat others instead of cooperate with them.

Regions identified as anatomical correlates of SFAM/CAM, such as OFC and the precuneus, have previously been identified as anatomical correlates of other cognitive factors. This may be because complex cognitive components are composed of multiple subcognitive components that are shared by multiple complex cognitive components. For example, the precuneus has a number of cognitive functions (Cavanna and Trimble 2006), and although this region has been identified as an anatomical correlate of CAM, it has also been identified as an anatomical correlate of other cognitive functions that are positively and negatively related to pro-social cognitive components (Banissy et al. 2012; Takeuchi et al. 2010b, 2011a). CAM is apparently negatively related to pro-social components, and this pro-social subcomponent may be shared by a number of distinct complex cognitive functions. Of course, sometimes the same region has multiple functions (Cavanna and Trimble 2006), adjacent regions have different functions (Kringelbach 2005), or one unified principle function of the region can appear as various seemingly different functions (Egner 2011). This may also lead to the same anatomical regions being identified as neural correlates of different cognitive components.

On the other hand, CAM and SFAM were associated with rather different structural brain characteristics. SFAM and CAM were psychologically derived through factor analysis, and thus although they are both part of achievement motivation, very little correlation was observed between the two (*r* = 0.24). Furthermore, as shown in the behavioral data of the “Results” section, their psychometric properties in terms of their association with personality measures were very different and distinctive. However, this may have been caused by the regressing out procedure in multiple regression analyses because the two factors were included as covariates in the same multiple regression analyses. We therefore performed two additional separate multiple regression analyses, one that included only SFAM and not CAM and another that included only CAM and not SFAM. These analyses did not change the significance of the study findings.

This study has a few limitations. One is common to our previous studies as well as other studies that use college cohorts (Jung et al. 2010; Song et al. 2008; Takeuchi et al. 2010a, b, 2011b). We used young healthy subjects with high educational backgrounds. Limited sampling of the full range of intellectual abilities is a common hazard when sampling from college cohorts (Jung et al. 2010). Whether our findings would also hold across the full range of population samples and a normal distribution must be determined with larger and more representative samples. However, in one sense, this may be less of an issue because as far as we are aware, the psychometric properties of achievement motivation do not differ between those with higher and lower intelligence, unlike creativity (Sternberg 2005). On the other hand, it may be important to focus on subjects who have grown up in an advantageous environment. This is because thinned cortices may represent advanced and thus adaptive cortical development. They may also stem from degenerated and thus maladaptive neuronal systems (Takeuchi et al. 2011a). Thus, removing subjects who did not attend college because of reasons such as substantial maldevelopment from the study sample may be important for preventing complicated non-linear associations between cognitions and GM structures.

This is the first study to investigate the association between rGM structures and achievement motivation. Previous functional imaging studies have investigated motivation-related brain activation modulated by external factors as well as the individual characteristics of motivation. Our study newly investigated anatomical correlates of two aspects of achievement motivation, SFAM and CAM. We found that structural variations in OFC underlie SFAM and those in the right insula, right putamen, and precuneus underlie CAM. These results suggest that the brain areas that play central roles in externally modulated motivation, namely OFC and the putamen, also contribute to SFAM and CAM, respectively. Furthermore, the brain areas involved in cognitive processes associated with distressing emotions and social cognition are associated with CAM.
